# Content and quality of clinical practice guidelines for the management of type 2 diabetes in India: A systematic review

**DOI:** 10.1002/edm2.405

**Published:** 2023-01-16

**Authors:** Oluwasegun P. Olujide, Mariama E. Olujide, Jo Leonardi‐Bee, Kaushik Chattopadhyay

**Affiliations:** ^1^ Lifespan and Population Health Academic Unit, School of Medicine University of Nottingham Nottingham UK; ^2^ Waziri Shehu Gidado General Hospital Kano Nigeria; ^3^ The Nottingham Centre for Evidence‐Based Healthcare: A JBI Centre of Excellence Nottingham UK

**Keywords:** clinical practice guideline, India, systematic review, type 2 diabetes mellitus

## Abstract

**Introduction:**

Over the last few decades, India has witnessed an increase in the number of people with type 2 diabetes mellitus (T2DM). Consequently, several clinical practice guidelines (CPGs) have been developed to assist western and traditional Indian medicine practitioners in managing this disease. This systematic review aimed to evaluate and synthesize the content and quality of these CPGs.

**Methods:**

Several databases and sources were searched from inception to May 2022, to identify CPGs for managing adults with T2DM in India. The screening of titles and abstracts and full texts, data extraction and quality assessment were conducted by two independent reviewers. Any disagreements were resolved through discussion or by involving a third reviewer. A data extraction tool from a previous study was adapted to extract the content of the included CPGs, and the Appraisal of Guidelines for Research and Evaluation II tool was used to assess the quality of the included CPGs. A narrative synthesis was conducted.

**Results:**

Of 3350 records identified, 11 were retrieved for full‐text screening and five CPGs were included in this systematic review—three focused on traditional Indian medicine (Ayurveda) and two focused on western medicine. These two western medicine CPGs contained comprehensive recommendations for managing T2DM but only one of these, the Research Society for the Study of Diabetes in India/Endocrine Society of India (RSSDI/ESI) CPG, was of high quality.

**Conclusions:**

Only one CPG can be recommended for managing T2DM by western medicine practitioners in India. Future CPGs, especially for traditional Indian medicine practitioners, should be developed and updated using the standard CPG manuals and quality appraisal tools.

**Registration:**

PROSPERO (CRD42021279499).

## Graphical abstract

In India, we found three clinical practice guidelines (CPGs) on traditional Indian medicine (Ayurveda) and two on western medicine. These two western medicine CPGs contain comprehensive recommendations for managing type 2 diabetes mellitus (T2DM) but only one of these, the Research Society for the Study of Diabetes in India/Endocrine Society of India (RSSDI/ESI) CPG, is of high quality. Only one CPG can be recommended for managing T2DM by western medicine practitioners in India. Future CPGs, especially for traditional Indian medicine practitioners, should be developed and updated using the standard CPG manuals and quality appraisal tools.

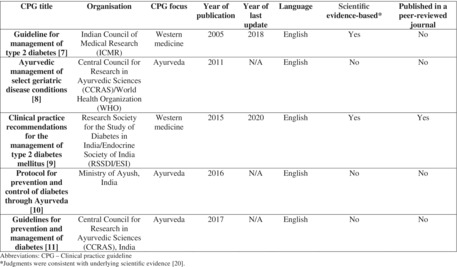

## 1 INTRODUCTION

India has approximately 74 million people with diabetes mellitus (DM),^1^ which is alarming, given the rapid rise in cases from 26 million in 1990.^2^ According to the International Diabetes Federation, over 90% of people with DM in India have type 2 diabetes mellitus (T2DM), and in 2021 alone, India witnessed about 650,000 DM‐related deaths and spent over $8.4 billion on DM‐related healthcare.^1^

To reduce morbidity, mortality and socioeconomic burden from T2DM, medical practitioners are advised to follow clinical practice guidelines (CPGs).^3^ CPGs contain recommendations to improve patient care that should be based on systematic reviews of the scientific literature and after a thorough assessment of the quality of the scientific evidence.^4^ The potential benefits of CPGs include improvement in health outcomes, consistency of healthcare delivery and enabling patients to make informed decisions about their condition.^4^, ^5^ Furthermore, high‐quality CPGs can improve healthcare professionals' clinical decision‐making and improve service delivery efficiency by discouraging the use of inefficient or ineffective services and procedures.^4^, ^5^ Conversely, a poorly developed CPG can inadvertently lead to the institutionalization of harmful clinical practices that may translate to adverse health outcomes.^4^, ^5^ Due to the extreme importance of CPGs, they must be rigourously developed using the best available scientific evidence.^4^

In India, western medicine (allopathy) and traditional Indian medicine such as Ayurveda are used to manage T2DM,^6^, ^7^ and several CPGs have been developed for medical practitioners to manage T2DM.^7^, ^8^, ^9^, ^10^, ^11^ However, to date, no systematic review has been conducted to assess the content and quality of CPGs for managing T2DM in India. Therefore, this systematic review aimed to evaluate and synthesize the content and quality of these CPGs. Evaluating the content of these CPGs showed if essential recommendations for managing T2DM were covered and the scientific evidence that underlies these recommendations. Relatedly, assessing the quality of these CPGs enabled the evaluation of methodological rigour in their development. The findings of this review could help improve the current CPGs and develop and update the future CPGs.

## 2 METHODS

The systematic review protocol was registered with PROSPERO (CRD42021279499) and reported using the Preferred Reporting Items for Systematic Review and Meta‐Analyses guidelines.^12^

### 2.1 Eligibility criteria

Western medicine and traditional Indian medicine (such as Ayurveda) CPGs that focused on the management of T2DM in adults (≥18 years) at all levels of healthcare in India were included. We included CPGs that were either scientific evidence‐based or non‐evidence‐based, published in peer‐reviewed journals or unpublished and national in scope. CPGs were included regardless of the language or date of publication. If a CPG was updated, the latest version was included. CPGs developed exclusively for managing T2DM in specific conditions, for example, in pregnancy, were excluded. CPGs under development, conference abstracts and commentaries were excluded.

### 2.2 Search strategy

The search strategies to identify CPGs were developed with assistance from a Senior Research Librarian at the University of Nottingham (Appendix S1). The following databases were searched from inception to 20th May 2022: MEDLINE (Ovid), Embase (Ovid), CINAHL (EBSCOhost), Web of Science (Clarivate), Scopus (Elsevier), PsycINFO (Ovid), Allied and Complementary Medicine Database (AMED) (Ovid), Turning Research Into Practice (TRIP), Guideline International Network (GIN), Guideline Central and Index Medicus for South‐East Asia Region (IMSEAR). In addition, the following national ministries and councils and major diabetes‐related societies in India were searched on 20th May 2022: Ministry of Health and Family Welfare (https://main.mohfw.gov.in/), Ministry of Ayush (https://www.ayush.gov.in/), Central Council for Research in Ayurvedic Sciences (CCRAS) (http://ccras.nic.in), Central Council for Research in Siddha (http://siddhacouncil.com/home/), Indian Council of Medical Research (ICMR) (https://www.icmr.gov.in/), Research Society for the Study of Diabetes in India (RSSDI) (https://www.rssdi.in/newwebsite/index.php) and Endocrine Society of India (ESI) (https://www.endocrinesocietyindia.org/). Furthermore, the reference lists of all the eligible CPGs were screened to identify other potentially eligible CPGs. We also searched the *Indian Journal of Endocrinology and Metabolism*, the *International Journal of Diabetes in Developing Countries* and the *Journal of the Association of Physicians* in India on 30th May 2022 as these journals were referenced in some of the eligible CPGs.

### 2.3 Screening process

All the retrieved records were aggregated and imported into EndNote X9.2 (Clarivate Analytics, PA, USA), and duplicates were removed. The titles and abstracts were screened, followed by the screening of full texts using the systematic review eligibility criteria by two independent reviewers (OPO and MEO). Any disagreements were resolved through discussion or by involving a third reviewer (KC). Those without an abstract were kept for full‐text screening. Full texts that did not meet the eligibility criteria were excluded, and the reasons for their exclusion are provided (Appendix S2). The organizations that developed the eligible CPGs were twice contacted via email to obtain the detailed development methodology and supplementary documents.

### 2.4 Data extraction

A data extraction tool from a previous study^13^ was adapted to extract the content (and related recommendations) of the included CPGs and conducted independently by two reviewers (OPO and MEO) with discrepancies resolved through discussion or with a third reviewer (KC). The broad content headings are as follows: blood glucose assessment and management, blood pressure assessment and management, body weight assessment and management, blood lipid assessment and management, assessment and management of T2DM‐related complications and other related issues and healthcare advice.

### 2.5 Quality assessment

The Appraisal of Guidelines for Research and Evaluation II (AGREE II) tool^14^ was used to assess the quality of the included CPGs. This tool contains 23 items grouped into six domains ((i) scope and purpose of the guideline, (ii) stakeholder involvement, (iii) rigour of development, (iv) clarity of presentation, (v) applicability and (vi) editorial independence) rated on a seven‐point Likert scale (ranging from 1—strongly disagree to 7—strongly agree).^14^ The tool also contains two global rating items: (i) overall assessment of guideline quality, rated on a seven‐point Likert scale and (ii) whether the guideline can be recommended for use in practice, and it is rated as ‘yes’, ‘yes with modification’ or ‘no’.^14^ The score for each domain was calculated using the formula detailed in the AGREE II manual.^14^ The appraisal was conducted independently by two reviewers (OPO and MEO) after completing the AGREE II online training module, with discrepancies resolved through discussion or with a third reviewer (KC).

The AGREE II tool does not stipulate a cut‐off to show if a domain is adequately addressed or not. However, in congruence with previous CPG appraisals,^15^, ^16^, ^17^ a domain was considered adequately addressed if a score of ≥60% was obtained. For assessing the overall quality, we considered a CPG to be of high quality (quality score of 5–7) if it achieved a score ≥60% in at least three domains, including domain 3 (rigour of development). CPGs that did not meet this criterion were considered to be of low quality (quality score of 1–4). Domain 3 is recognized as the strongest indicator of CPG quality, and a high score indicates a scientific evidence‐based CPG development and minimum bias.^18^

Only high‐quality CPGs that adequately addressed domain 4 (clarity of presentation) were recommended for use without modifications. Low‐quality CPGs were recommended with modifications only if they adequately addressed at least three domains, including domain 4. The reliance on domain 4 for recommending a CPG for use was based on findings of a systematic review.^19^

### 2.6 Data synthesis

A narrative synthesis was conducted. The characteristics of the included CPGs were summarized, followed by a narrative synthesis based on identifying patterns within the results of the CPGs and comparing and contrasting the findings between CPGs focusing on western medicine and those on traditional Indian medicine.

## 3 RESULTS

### 3.1 Literature search

Of 3350 records identified, 897 were duplicates, 2441 were ineligible (2429 titles and abstracts were unrelated to CPG, one was a CPG under development, four were conference abstracts and seven were commentaries) and 11 were retrieved for full‐text screening. Five CPGs were included in this systematic review^7^, ^8^, ^9^, ^10^, ^11^ (Appendix S3), and the six excluded at the full‐text screening stage were due to not being a CPG (*n* = 1), not being a T2DM‐specific CPG (*n* = 3) or being an older version of an included CPG (*n* = 2).

### 3.2 Characteristics of included CPGs

The characteristics of all five CPGs are shown in Table 1. The included CPGs were published or last updated between 2011 and 2020 and written in the English language.^7^, ^8^, ^9^, ^10^, ^11^ Three of the included CPGs focused on Ayurveda,^8^, ^10^, ^11^ and the remaining two focused on western medicine.^7^, ^9^ A scientific evidence‐based approach was used to develop the western medicine CPGs,^7^, ^9^ and one of these was published in a peer‐reviewed journal.^9^

**TABLE 1:** Summary of the included CPGs for managing T2DM in India

CPG title	Organization	CPG focus	Year of publication	Year of last update	Language	Scientific evidence‐based^a^	Published in a peer‐reviewed journal
Guideline for management of type 2 diabetes^7^	Indian Council of Medical Research (ICMR)	Western medicine	2005	2018	English	Yes	No
Ayurvedic management of select geriatric disease conditions^8^	Central Council for Research in Ayurvedic Sciences (CCRAS)/World Health Organization (WHO)	Ayurveda	2011	N/A	English	No	No
Clinical practice recommendations for the management of type 2 diabetes mellitus^9^	Research Society for the Study of Diabetes in India/Endocrine Society of India (RSSDI/ESI)	Western medicine	2015	2020	English	Yes	Yes
Protocol for prevention and control of diabetes through Ayurveda^10^	Ministry of Ayush, India	Ayurveda	2016	N/A	English	No	No
Guidelines for prevention and management of diabetes^11^	Central Council for Research in Ayurvedic Sciences (CCRAS), India	Ayurveda	2017	N/A	English	No	No

Abbreviations: CPG, Clinical practice guideline; T2DM, type 2 diabetes mellitus.

^a^ Judgements were consistent with underlying scientific evidence.^20^

### 3.3 Content of CPGs

#### 3.3.1 Blood glucose assessment and management

All the CPGs^7^, ^8^, ^9^, ^10^, ^11^ contained recommendations for the assessment of T2DM (Table 2). For the diagnosis of T2DM, three CPGs^7^, ^9^, ^10^ recommended any one of the following criteria: fasting plasma glucose ≥126 mg/dl, 2‐h plasma glucose ≥200 mg/dl, glycated haemoglobin (HbA1c) ≥6.5% (48 mmol/mol) and random plasma glucose ≥200 mg/dl in the presence of diabetes symptoms. The CCRAS CPG^11^ recommended a fasting plasma glucose ≥126 mg/dl and random plasma glucose ≥200 mg/dl in the presence of diabetes symptoms, while the CCRAS/World Health Organization (WHO) CPG^8^ recommended the use of 2‐h plasma glucose ≥200 mg/dl in addition to the two criteria contained in CCRAS CPG.^11^ Recommendations for topics related to the management of blood glucose were included in all the CPGs to varying degrees. The CPGs on western medicine^7^, ^9^ contained information on Ayurveda but did not recommend it for the management of T2DM.

**TABLE 2:** Content of the included CPGs for managing T2DM in India

Content		CPGs				
		RSSDI/ESI^9^	ICMR^7^	CCRAS^11^	Ministry of Ayush^10^	CCRAS/WHO^8^
Blood glucose assessment and management	T2DM diagnosis	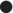	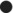	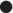	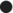	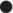
	Blood glucose target	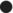	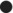	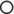	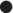	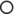
	Self‐monitoring of blood glucose	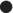	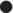	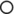	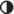	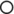
	Postprandial hyperglycaemia	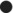	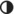	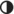	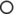	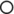
	T2DM self‐management education	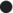	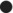	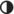	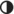	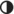
	Healthy diets	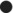	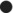	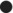	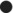	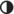
	Medical nutrition therapy (tailored diet)	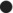	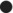	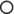	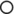	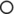
	Physical activity	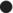	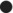	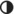	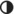	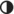
	Smoking cessation	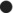	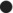	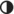	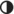	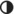
	Reduction in alcohol consumption	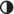	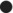	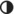	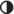	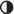
	Ayurveda	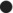	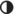	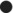	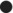	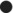
	Monotherapy^a^	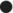	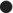	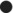	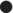	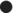
	Dual therapy^b^	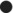	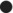	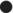	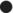	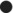
	Triple therapy^c^	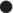	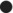	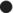	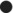	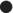
	Insulin therapy (insulin alone)	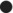	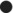	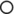	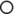	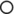
Blood pressure assessment and management	Blood pressure measurement and targets	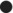	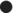	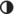	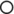	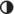
	Antihypertensive treatment	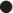	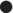	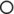	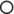	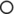
Body weight assessment and management	Body mass index (BMI) measurement	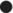	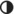	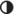	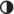	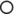
	Waist circumference measurement	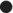	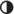	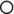	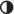	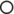
	Anti‐obesity drugs	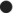	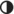	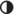	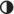	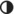
	Bariatric surgery	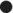	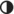	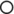	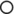	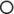
Blood lipid assessment and management	Blood lipids measurement	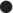	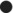	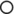	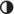	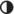
	Lipid‐lowering drugs	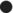	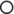	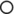	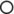	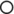
Assessment and management of T2DM‐related complications	Hyperosmolar hyperglycaemic state	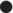	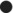	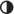	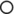	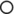
	Diabetic ketoacidosis	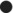	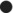	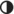	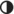	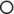
	Hypoglycaemia	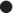	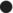	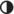	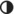	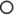
	Diabetic nephropathy	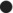	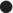	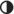	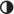	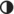
	Diabetic retinopathy	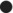	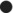	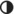	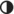	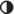
	Diabetic neuropathy	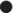	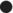	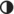	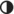	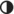
	Diabetic foot syndrome	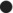	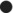	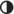	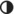	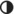
	Cardiovascular diseases	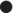	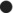	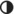	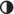	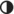
	Peripheral vascular disease	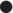	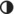	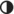	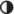	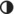
	Mental health	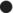	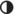	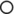	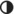	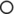
Other related issues and healthcare advice	Infectious diseases (e.g. respiratory and urinary tract infections)	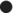	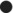	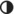	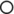	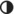
	Immunization against infectious diseases (e.g. pneumococcal and influenza vaccines)	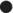	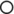	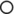	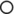	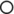
	Older people	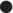	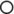	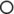	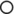	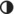
	Critical illnesses	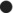	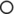	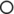	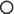	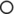
	Referral to specialists	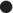	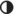	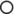	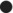	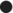
	Fasting (e.g. for religious purposes)	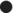	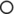	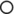	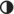	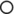
	Driving	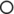	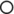	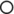	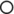	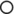
	Pregnancy	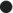	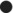	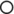	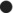	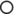
	Surgery	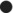	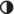	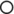	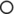	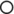
	Travel	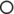	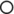	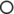	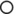	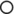
	Health insurance	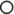	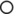	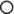	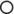	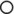
	Technologies for T2DM management^d^	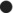	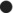	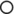	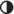	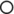

Abbreviations: AGREE II, Appraisal of Guidelines for Research and Evaluation II; CCRAS, Central Council for Research in Ayurvedic Sciences; T2DM, Type 2 diabetes mellitus; CPG, Clinical practice guideline; ICMR, Indian Council of Medical Research; RSSDI/ESI, Research Society for the Study of Diabetes in India/Endocrine Society of India; WHO, World Health Organization.

*Note*: 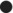, Detailed information on the topic or topic presented as a heading or sub‐heading in the CPG. 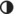, Limited information on the topic in the CPG. 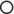, No information on the topic in the CPG.

^a^ Monotherapy is the initial treatment regimen with one oral drug (western or Ayurvedic medicine).

^b^ Dual therapy is the addition of a second drug (western or Ayurvedic medicine) when the initial drug is insufficient to reduce blood glucose to recommended levels.

^c^ Triple therapy is the addition of a third drug (western or Ayurvedic medicine) when dual therapy is insufficient to reduce blood glucose to recommended levels.

^d^ Examples include blood glucose meters, insulin pumps and insulin pens.

#### 3.3.2 Blood pressure assessment and management

Only CPGs on western medicine^7^, ^9^ contained recommendations for the assessment of blood pressure. They recommended regular blood pressure monitoring with a target of <140/90 mmHg in all patients with T2DM and a target of <130/80 mmHg in patients with T2DM at a higher risk of chronic kidney disease.^7^, ^9^ While CPGs on western medicine^7^, ^9^ outlined both pharmacological and non‐pharmacological approaches for the reduction of blood pressure, Ayurvedic CPGs^8^, ^10^, ^11^ did not contain such recommendations.

#### 3.3.3 Body weight assessment and management

For the assessment of body weight, only the CPGs on western medicine^7^, ^9^ contained recommendations for cut‐offs to define overweight and obesity. The RSSDI/ESI CPG^9^ defined normal weight as a body mass index (BMI) of 18–22.9 kg/m^2^, overweight as a BMI of 23–24.9 kg/m^2^ and obesity as a BMI ≥25 kg/m^2^. The ICMR CPG^7^ recommended slightly different cut‐offs and defined normal weight as a BMI of 20–23 kg/m^2^, overweight as a BMI of 23.1–25 kg/m^2^ and obesity as a BMI >25 kg/m^2^. In addition to lifestyle modifications, all the CPGs^7^, ^8^, ^9^, ^10^, ^11^ contained specific recommendations for anti‐obesity medications. However, only the RSSDI/ESI CPG^9^ provided detailed recommendations for bariatric surgery.

#### 3.3.4 Blood lipids assessment and management

CPGs on western medicine^7^, ^9^ contained information on the recommended levels of blood lipids in patients with T2DM. The rest of the CPGs^8^, ^10^, ^11^ were devoid of this information. Only the RSSDI/ESI CPG^9^ recommended medications for lowering blood lipids.

#### 3.3.5 Assessment and management of T2DM‐related complications

Although CPGs on western medicine^7^, ^9^ contained information on all three acute complications, only the RSSDI/ESI CPG^9^ provided specific recommendations for their assessment. Ayurvedic CPGs^8^, ^10^, ^11^ contained no recommendations for acute complications of T2DM. Recommendations for the assessment of chronic complications of T2DM were mentioned in two CPGs.^7^, ^9^ Recommendations for the management of complications were found in three CPGs.^7^, ^9^, ^10^

#### 3.3.6 Other related issues and healthcare advice

To varying degrees, all the CPGs^7^, ^8^, ^9^, ^10^, ^11^ included recommendations for other related issues and healthcare advice. Only one CPG^9^ provided recommendations for most of the topics in this domain.

### 3.4 Quality of CPGs

#### 3.4.1 Domain scores

‘Scope and purpose’ and ‘clarity of presentation’ were the highest‐scoring domains with mean scores of 64% and 74%, respectively. As reported in Table 3, three of the CPGs^7^, ^9^, ^10^ adequately addressed (i.e. scored ≥60%) these domains. With a mean score of 47%, the ‘stakeholder involvement’ domain was adequately addressed by the CPGs on western medicine.^7^, ^9^ ‘Rigour of development’ and ‘editorial independence’ domains jointly scored lowest with a mean score of 24%, and only the RSSDI/ESI CPG^9^ adequately addressed both domains. The ‘applicability’ domain which had a mean score of 40% was adequately addressed by the RSSDI/ESI CPG.^9^

**TABLE 3:** AGREE II domain scores and overall assessment of CPGs for managing T2DM in India

CPGs	AGREE II domains						Overall CPG assessment	
	1	2	3	4	5	6		
	Scope and purpose (%)	Stakeholder involvement (%)	Rigour of development (%)	Clarity of presentation (%)	Applicability (%)	Editorial independence (%)	Overall quality	Recommended for use in practice
RSSDI/ESI^9^	97	91	63	100	79	75	6.75	‘Yes’
ICMR^7^	81	62	19	83	38	21	4	‘Yes, with modifications’
CCRAS^11^	22	3	17	61	21	0	2	‘No’
Ministry of Ayush^10^	78	32	17	81	42	4	3	‘No’
CCRAS/WHO^8^	42	47	5	44	19	20	2	‘No’

Abbreviations: AGREE II, Appraisal of Guidelines for Research and Evaluation II; CCRAS, Central Council for Research in Ayurvedic Sciences; CPG, Clinical practice guideline; ICMR, Indian Council of Medical Research; RSSDI/ESI, Research Society for the Study of Diabetes in India/Endocrine Society of India; WHO, World Health Organization.

#### 3.4.2 Overall CPG assessment

The overall CPG quality ratings ranged from 2 to 6.75 out of 7. The RSSDI/ESI CPG^9^ sufficiently addressed all the domains and had a quality rating of 6.75. This high‐quality CPG was recommended for use without modification.

## 4 DISCUSSION

This systematic review assessed and synthesized the content and quality of five CPGs^7^, ^8^, ^9^, ^10^, ^11^ for managing T2DM by medical practitioners in India. Two of the CPGs focused on western medicine,^7^, ^9^ and three focused on the use of Ayurveda.^8^, ^10^, ^11^ There were significant variations in recommendations between the two groups of CPGs and within the CPGs, which could lead to unacceptable variations in clinical practice. In addition, multiple CPGs for managing T2DM in a given context might confuse the medical practitioners and thus, a single CPG for each system of medicine will be helpful. Medical practitioners in India can access international CPGs; however, local CPGs are important as contextualized judgements are made in guidelines.^9^

CPGs on western medicine^7^, ^9^ provided more comprehensive recommendations. The RSSDI/ESI CPG^9^ contained scientific evidence‐based recommendations comparable to other high‐quality international guidelines.^13^ In the CCRAS/WHO CPG published in 2011,^8^ the recommendations might have been compiled before the WHO approved the usage of HbA1c to diagnose T2DM in the same year.^21^ The CCRAS CPG^11^ was published in 2017, and the omission likely reflects a failure to review the current literature as the outdated WHO recommendation was used. This omission could negatively impact clinical practice, as HbA1c is the most accurate method to assess long‐term blood glucose control for preventing T2DM complications.^22^ The blood pressure targets in patients with T2DM mentioned in western medicine CPGs^7^, ^9^ are consistent with the recommendations in high‐quality international CPGs.^22^, ^23^, ^24^ The recommendations in these CPGs^7^, ^9^ for the usage of angiotensin‐converting enzyme inhibitors or angiotensin receptor blockers to reduce blood pressure reflect the current evidence of their effectiveness and reno‐protective effects.^25^, ^26^ The recommendations for body weight assessment contained in the RSSDI/ESI CPG^9^ were developed by a consensus group in India,^27^ and it mirrors the lower BMI cut‐offs recommended by WHO for people residing in the Asia‐Pacific region.^28^ The ICMR CPG's^7^ divergence from these recommended cut‐offs could be due to the specific recommendations provided by its guideline development group. Until evidence is provided to support the ICMR CPG's^7^ recommendation, its use will remain contentious. Furthermore, the RSSDI/ESI CPG's^9^ recommendations for medications and bariatric surgery are consistent with the current evidence.^22^, ^23^ The recommendations for blood lipid targets provided in western medicine CPGs^7^, ^9^ reflect those outlined in a consensus statement for managing dyslipidaemia in Indians with T2DM.^29^ The recommendations for medications to manage dyslipidaemia are also consistent with the current evidence.^22^, ^23^ The recommendations for assessing and managing T2DM‐related complications provided in western medicine CPGs^7^, ^9^ are consistent with those found in other high‐quality international CPGs.^22^, ^23^ Ayurvedic CPGs^8^, ^10^, ^11^ lacked recommendations for most of the T2DM‐related complications, and this is presumably because Ayurvedic practitioners need to refer emergencies and most complicated cases for allopathic treatment.^8^, ^10^

‘Scope and purpose’ and ‘clarity of presentation’ domains were adequately addressed by three CPGs,^7^, ^9^, ^10^ and the high scores are consistent with findings in other studies.^30^, ^31^ These high scores might be attributed to the ease of adequately addressing these domains or a greater emphasis placed on these domains by CPG developers.^30^ CPGs scored poorly in the ‘stakeholder involvement’ and ‘rigour of development’ domains, and the findings are similar to those in other studies.^30^, ^31^ The first one is a failure to appreciate the relevance of stakeholders' and patients' views and preferences in CPG development.^32^ The prohibitive cost of assembling all the relevant stakeholders or poor reporting of stakeholder involvement in CPG development could be other reasons.^32^ In Ayurvedic CPGs,^8^, ^10^, ^11^ the scientific rigour in the guideline development process was lacking. The failure of CPG developers to appreciate the importance of reporting on the sources and quality of scientific evidence used in CPG development could be a major issue. The ‘applicability’ and ‘editorial independence’ domains were adequately addressed by the RSSDI/ESI CPG,^9^ and the findings are consistent with those in other studies.^31^, ^33^ This suggests that CPG developers did not give much attention to the applicability and resource implications of implementing CPG recommendations. An apparent disregard for conflict of interest disclosures by CPG developers or the actual existence of a conflict of interest between CPG developers and funding sources could be a major issue.^34^, ^35^ Overall, the failure to adequately address all of these domains could negatively impact the trustworthiness and implementation of CPGs.^4^, ^32^ The RSSDI/ESI CPG,^9^ which had a score of 6.75 and was recommended in this systematic review, was as good as CPGs from England, Scotland, Canada and the United States with quality scores of 7, 7, 6 and 5, respectively.^13^ The rest of the CPGs included in this systematic review fell short.

### 4.1 Strengths and limitations of the systematic review

A robust systematic review process was followed. An extensive search of a range of sources was conducted without language or date limitation, thus making the omission of potentially eligible CPGs highly unlikely. The AGREE II tool,^14^ the most widely used tool for evaluating CPG quality, was used to appraise the CPGs. The appraisal was conducted by two independent reviewers (OPO and MEO) who are medical practitioners and underwent training in using the tool. There was an excellent level of reliability between them (intraclass correlation coefficient >0.9). While the AGREE II tool^14^ focuses on the methodological process of CPG development, it does not assess the quality of the scientific evidence that underpins the CPG. A consequence of this is the inability of this systematic review to state the true strength or weakness of some of the CPGs' recommendations to medical practitioners. We aimed to overcome the effect of this limitation by analysing the contents of the included CPGs; unfortunately, none of the included CPG mentioned the levels of scientific evidence or grades of recommendations and therefore, this limitation could not be attenuated. Another limitation posed using the AGREE II tool is that it lacked specific guidance on conducting the overall assessment of CPG quality. However, this review used a popular criterion to enable comparison with other similar studies. The possibility that CPGs' quality might differ depending on the method used to ascertain their overall quality presents a level of subjectivity in reporting, and this review must be considered in that light.

### 4.2 Recommendations for clinical practice

The RSSDI/ESI CPG^9^ has the greatest potential to assist medical practitioners (western medicine) in managing T2DM.

### 4.3 Recommendations for CPG development

Future CPGs, especially for traditional Indian medicine practitioners such as Ayurveda,^36^ should be developed and updated using the standard CPG manuals and quality appraisal tools, after taking into consideration the best available scientific evidence. The CPGs should cover all the areas of T2DM management.

### 4.4 Recommendations for future research

Reviewers should give more weight to the ‘rigour of development’ domain when assessing the overall quality of CPGs until the AGREE Enterprise provides specific guidance. This domain contains the majority of items and is recognized as the strongest indicator of quality. The usage of this domain to underpin quality can ensure the consistency of future reviews and enable direct comparison of findings.

## 5 CONCLUSION

Only one CPG can be recommended for managing T2DM by western medicine practitioners in India. Future CPGs, especially for traditional Indian medicine practitioners, should be developed and updated using the standard CPG manuals and quality appraisal tools.

## AUTHOR CONTRIBUTIONS

KC and OPO conceptualized and designed the systematic review. OPO, MEO and KC conducted the systematic review. OPO wrote the first draft of the manuscript with the support of KC. MEO, JLB and KC contributed significantly to the revision of the manuscript and read and approved the final manuscript.

## CONFLICT OF INTEREST

None.

## Supporting information


Appendix S1–S3

## Data Availability

Data sharing is not applicable to this article as no new data were created or analyzed in this study.
