# Supplementary material for: Content and quality of clinical practice guidelines for the management of type 2 diabetes in India: A systematic review
Source: Endocrinol Diabetes Metab. 2023 Jan 16;6(2):e405. doi: 10.1002/edm2.405 (PMC10000656; doi:10.1002/edm2.405)
Supplement: Supplementary file 1 — Appendix S1–S3 [file EDM2-6-e405-s001.docx]

**SUPPLEMENTARY MATERIALS**

##

## Appendix 1. Search strategies

### MEDLINE (Ovid)

Date: 1946 – 20^th^ May 2022

Hits: 290

| **Search number** | **Search term** | **Results** |
| --- | --- | --- |
| 1. | exp Practice Guideline/ | 28,812 |
| 2. | ("practice guideline*" or guideline* or "clinical guideline*" or "clinical practice guideline* practice guideline as topic" or recommendation* or CPG or CPGs).mp. | 758,801 |
| 3. | exp Consensus/ | 15,785 |
| 4. | exp Evidence-Based Practice/ | 91,019 |
| 5. | exp Practice Guidelines as Topic/ | 124,439 |
| 6. | (consensus or "evidence-based practice*" or "practice guideline as topic").mp. | 215,751 |
| 7. | 1 or 2 or 3 or 4 or 5 or 6 | 975,579 |
| 8. | exp Diabetes Mellitus, Type 2/ | 144,011 |
| 9. | (Type* adj3 ("2" or "II" or two*) adj3 (diabete* or diabetic*)).mp. | 208,097 |
| 10. | ((Late or maturit* or adult* or slow*) adj3 onset* adj3 (diabete* or diabetic*)).mp. | 3,447 |
| 11. | ((Ketosis-resistant* or stable*) adj3 (diabete* or diabetic*)).mp. | 820 |
| 12. | ((Non-insulin* or Non insulin* or Noninsulin*) adj3 depend* adj3 (diabete* or diabetic*)).mp. | 11,959 |
| 13. | (NIDDM or T2DM or T2D or prameha or madhumeha).mp. | 43,330 |
| 14. | 8 or 9 or 10 or 11 or 12 or 13 | 214,268 |
| 15. | exp India/ | 109,524 |
| 16. | (India* or Bharat* or Hindustan*).mp. | 233,536 |
| 17. | 15 or 16 | 233,566 |
| 18. | 7 and 14 and 17 | 290 |

### Embase (Ovid)

Date: 1974 – 20^th^ May 2022

Hits: 704

| **Search number** | **Search term** | **Result** |
| --- | --- | --- |
| 1. | exp practice guideline/ | 603328 |
| 2. | ("practice guideline*" or guideline* or "clinical guideline*" or "clinical practice guideline* practice guideline as topic" or recommendation* or CPG or CPGs).mp. | 1176507 |
| 3. | exp consensus development/ or exp consensus | 104125 |
| 4. | exp evidence based practice/ | 1462147 |
| 5. | (consensus or “consensus development” or “evidence based practice*”) | 350959 |
| 6. | 1 or 2 or 3 or 4 or 5 | 2737641 |
| 7. | exp non insulin dependent diabetes mellitus/ | 278890 |
| 8. | (Type* adj3 ("2" or "II" or two*) adj3 (diabete* or diabetic*)).mp. | 255182 |
| 9. | ((Late or maturit* or adult* or slow*) adj3 onset* adj3 (diabete* or diabetic*)).mp. | 5121 |
| 10. | ((Ketosis-resistant* or stable*) adj3 (diabete* or diabetic*)).mp. | 1277 |
| 11. | ((Non-insulin* or Non insulin* or Noninsulin*) adj3 depend* adj3 (diabete* or diabetic*)).mp. | 282091 |
| 12. | (NIDDM or T2DM or T2D or prameha or madhumeha).mp. | 73267 |
| 13. | 7 or 8 or 9 or 10 or 11 or 12 | 346028 |
| 14. | exp “Punjab (India)”/ or exp India/ | 158589 |
| 15. | (“Punjab (India)” or India or Bharat* or Hindustan*).mp. | 229419 |
| 16. | 14 or 15 | 232246 |
| 17. | 6 and 13 and 16 | 704 |

**CINAHL (EBSCOhost)**

Date: 1961 – 20^th^ May 2022

Hits: 86

| **Search number** | **Search term** | **Results** |
| --- | --- | --- |
| S15 | S4 AND S11 AND S14 | 89 |
| S14 | S12 OR S13 | 71,117 |
| S13 | (India* or Bharat* or Hindustan*) | 71,117 |
| S12 | (MH "India") | 40,976 |
| S11 | S5 OR S6 OR S7 OR S8 OR S9 OR S10 | 89,781 |
| S10 | (NIDDM or T2DM or T2D or prameha or madhumeha) | 56,528 |
| S9 | ""((Non-insulin* or "Non insulin*" or Noninsulin*) N3 depend* N3 (diabete* or diabetic*))"" | 1,521 |
| S8 | ((Ketosis-resistant* or stable*) N3 (diabete* or diabetic*)) | 278 |
| S7 | ""((Late or maturit* or adult* or slow*) N3 onset* N3 (diabete* or diabetic*))"" | 4,498 |
| S6 | ""(Type* N3 ("2" or "II" or two*) N3 (diabete* or diabetic*))"" | 86,102 |
| S5 | (MH "Diabetes Mellitus, Type 2") | 67,207 |
| S4 | S1 OR S2 OR S3 | 354,702 |
| S3 | """practice guideline*" OR guideline* OR "clinical guideline*" OR "clinical practice guideline*" OR "practice guideline as topic" OR recommendation* OR "evidence based practice*" OR CPG OR CPGs"" | 351,161 |
| S2 | (MH "Consensus") | 6,055 |
| S1 | (MH "Practice Guidelines") | 83,484 |

### Web of Science (Clarivate)

Date: 1900 – 20^th^ May 2022

Hits: 639

| **Search number** | **Search term** | **Results** |
| --- | --- | --- |
| 1. | ((((((((((ALL=("practice guideline*")) OR ALL=(guideline*)) OR ALL=("clinical guideline*")) OR ALL=("clinical practice guideline*")) OR ALL=("practice guideline as topic*")) OR ALL=(recommendation*)) OR ALL=(CPG)) OR ALL=(CPGs)) OR ALL=(consensus)) OR ALL=("consensus development*")) OR ALL=("evidence based practice*") | 1,412,489 |
| 2. | (((ALL=("type 2 diabetes mellitus")) OR ALL=(T2DM)) OR ALL=(T2D)) OR ALL=(NIDDM) | 97,095 |
| 3. | TS=((Type* NEAR ("2" or "II" or two*) NEAR (diabete* or diabetic*))) | 215,414 |
| 4. | TS=(((Late or maturit* or adult* or slow*) NEAR onset* NEAR (diabete* or diabetic*))) | 4,547 |
| 5. | TS=(((Ketosis-resistant* or stable*) NEAR (diabete* or diabetic*))) | 2,789 |
| 6. | TS=(((Non-insulin* or "Non insulin*" or Noninsulin*) NEAR depend* NEAR (diabete* or diabetic*))) | 11,815 |
| 7. | ((((#2) OR #3) OR #4) OR #5) OR #6 | 236,542 |
| 8. | ((ALL=(India*)) OR ALL=(Bharat*)) OR ALL=(Hindustan*) | 2,842,643 |
| 9. | ((#1) AND #7) AND #8 | 639 |

###

### Scopus (Elsevier)

Date: 1970 – 20^th^ May 2022

Hits: 434

| **Search number** | **Search term** | **Results** |
| --- | --- | --- |
| 1. | (TITLE-ABS-KEY("practice guideline*" OR guideline* OR "clinical guideline*" OR "clinical practice guideline*" OR "practice guideline as topic*" OR recommendation* OR CPG OR CPGs OR consensus OR "consensus development*" OR "evidence based practice*")) AND ((TITLE-ABS-KEY("type 2 diabetes mellitus" OR "diabetes mellitus type 2" OR T2DM OR T2D OR NIDDM OR prameha OR madhumeha)) OR (TITLE-ABS-KEY(Type* W/3 ("2" or "II" or two*) W/3 (diabete* or diabetic*))) OR (TITLE-ABS-KEY((Late or maturit* or adult* or slow*) W/3 onset* W/3 (diabete* or diabetic*))) OR (TITLE-ABS-KEY((Ketosis-resistan* or stable*) W/3 (diabete* or diabetic*))) OR (TITLE-ABS-KEY((Non-insulin* or "Non insulin*" or Noninsulin*) W/3 depend* W/3 (diabete* or diabetic*)))) AND (TITLE-ABS-KEY(India* OR Bharat* OR Hindustan*)) | 434 |

###

### PsycINFO (Ovid)

Date: 1806 – 20^th^ May 2022

Hits: 19

| **Search number** | **Search term** | **Results** |
| --- | --- | --- |
| 1. | exp Treatment Guidelines/ | 7,840 |
| 2. | exp Evidence Based Practice/ | 19,121 |
| 3. | ("treatment guideline*" or "evidence based practice*" or "practice guideline*" or guideline* or "clinical guideline*" or "clinical practice guideline*” or “practice guideline as topic" or recommendation* or CPG or CPGs or consensus or "consensus development*").mp. | 223,918 |
| 4. | 1 or 2 or 3 | 223,918 |
| 5. | exp Type 2 Diabetes/ | 5,042 |
| 6. | (Type* adj3 ("2" or "II" or two*) adj3 (diabete* or diabetic*)).mp. | 8,978 |
| 7. | ((Late or maturit* or adult* or slow*) adj3 onset* adj3 (diabete* or diabetic*)).mp. | 91 |
| 8. | ((Ketosis-resistant* or stable*) adj3 (diabete* or diabetic*)).mp. | 19 |
| 9. | ((Non-insulin* or "Non insulin*" or Noninsulin*) adj3 depend* adj3 (diabete* or diabetic*)).mp. | 275 |
| 10. | (NIDDM or T2DM or T2D or prameha or madhumeha).mp. | 1.787 |
| 11. | 5 or 6 or 7 or 8 or 9 or 10 | 10,308 |
| 12. | (India* or Bharat* or Hindustan*).mp. | 43,922 |
| 13. | 4 and 11 and 12 | 19 |

### Allied and Complementary Medicine database (AMED) (Ovid)

Date: 1985 – 20^th^ May 2022

Hits: 2

| **Search number** | **Search term** | **Results** |
| --- | --- | --- |
| 1. | exp practice guidelines/ | 427 |
| 2. | exp Guidelines/ | 2,507 |
| 3. | exp Evidence based medicine/ | 3,047 |
| 4. | ("practice guideline*" or guideline* or "evidence based medicine*" or "clinical guideline*" or "clinical practice guideline*" or "practice guideline as topic*" or recommendation* or CPG or CPGs).mp. | 12,844 |
| 5. | (consensus or "consensus development*").mp. | 1,950 |
| 6. | 1 or 2 or 3 or 4 or 5 | 14,249 |
| 7. | exp diabetes mellitus type 2/ | 761 |
| 8. | (Type* adj3 ("2" or "II" or two*) adj3 (diabete* or diabetic*)).mp. | 1,290 |
| 9. | ((Late or maturit* or adult* or slow*) adj3 onset* adj3 (diabete* or diabetic*)).mp. | 16 |
| 10. | ((Ketosis-resistant* or stable*) adj3 (diabete* or diabetic*)).mp. | 3 |
| 11. | ((Non-insulin* or Non insulin* or Noninsulin*) adj3 depend* adj3 (diabete* or diabetic*)).mp. | 91 |
| 12. | (NIDDM or T2DM or T2D or prameha or madhumeha).mp. | 246 |
| 13. | 7 or 8 or 9 or 10 or 11 or 12 | 1,394 |
| 14. | exp India/ | 1,311 |
| 15. | (India* or Bharat* or Hindustan*).mp. | 2,550 |
| 16. | 14 or 15 | 2,550 |
| 17. | 6 and 13 and 16 | 2 |

**Transforming Research into Practice (TRIP)**

Date: Searched on 20^th^ May 2022

Hits: 892

Screened all the citations retrieved upon searching “diabetes”. Search was limited to “other – guidelines”.

**Guideline International Network (GIN)**

Date: Searched on 20^th^ May 2022

Hits: 1

Screened all the citations retrieved upon searching “diabetes”. Search was limited to “India”.

**Guideline Central (GC)**

Date: Searched on 20^th^ May 2022

Hits: 232

Screened all the citations retrieved upon searching “diabetes”.

**Index Medicus for South-East Asia Region (IMSEAR)**

Date: Searched on 20^th^ May 2022

Hits: 45

Screened all the citations retrieved upon searching “diabetes”. Search was limited by “Diabetes mellitus”, “Adult”, “Humans”, “Practice guidelines”.

## Appendix 2. Excluded CPGs with reasons for exclusion

| **Title** | **Organisation/ Author** | **Date of publication** | **Reasons for exclusion** |
| --- | --- | --- | --- |
| Consensus evidence-based guidelines for in-patient management of hyperglycaemia in non-critical care setting as per Indian clinical practice | Gangopadhyay K. et al | 2014 | Not a T2DM specific CPG. |
| Clinical practice recommendations for management of type 2 diabetes 2015 | Research Society for the Study of Diabetes in India (RSSDI) | 2015 | Older version of a current CPG. |
| RSSDI clinical practice recommendations for management of in-hospital hyperglycaemia | Research Society for the Study of Diabetes in India (RSSDI) | 2016 | Not a T2DM specific CPG. |
| Madhumeha (diabetes mellitus) | Ministry of Health and Family Welfare (MoHFW) | 2016 | Not a CPG. |
| Clinical practice recommendations for management of type 2 diabetes 2017 | Research Society for the Study of Diabetes in India (RSSDI) | 2017 | Older version of a current CPG. |
| Integration of Ayush with national program for prevention and control of cancer, diabetes, cardiovascular diseases, and stroke | Central Council for Research in Ayurvedic Sciences (CCRAS)/ Directorate General of Health Services | 2018 | Not a T2DM specific CPG. |

Abbreviations: CPG – Clinical practice guideline; T2DM – Type 2 diabetes mellitus

**Appendix 3. PRISMA flow diagram for systematic reviews**

**Identification of CPGs via other methods**

**Identification of CPGs via databases**

Records identified from databases

(n = 3344):

Medline (n = 290)

Embase (n = 704)

CINAHL (n = 86)

PsycINFO (n = 19)

Scopus (n = 434)

Web of Science (n = 639)

AMED (n = 2)

TRIP (n = 892)

GIN (n = 1)

Guideline Central (n = 232)

IMSEAR (n = 45)

Records removed before screening:

Duplicate records removed

(n = 897)

Records identified from:

Websites (n = 6)

**Identification**

Records (title and abstract) excluded

(n = 2441)

Records (title and abstract) screened

(n = 2447)

Reports not retrieved

(n = 0)

Reports sought for retrieval

(n = 6)

Reports sought for retrieval

(n = 6)

**Screening**

Reports not retrieved

(n = 1)

Reports (full-text) excluded:

Not a CPG (n = 1)

Not a T2DM specific CPG

(n = 1)

Reports (full-text) excluded:

Older edition of a current CPG (n = 2)

Not a T2DM specific CPG

(n = 2)

Reports (full-text) assessed for eligibility

(n = 5)

Reports (full-text) assessed for eligibility

(n = 6)

CPGs included in review

(n = 5)

**Included**
